# Linking mHealth Engagement to Depression, Anxiety, and Stress Through Perceived Health Competence

**DOI:** 10.3390/healthcare14030338

**Published:** 2026-01-29

**Authors:** Iliana-Carmen Bușneag, Alina Chiracu, Marilena Cojocaru, Adin Marian Cojocaru, Germina-Alina Cosma, Marian-Alexandru Cosma

**Affiliations:** 1 Faculty of Physical Education and Sport, University Spiru Haret, 030045 Bucharest, Romania; busneag.iliana@spiruharet.ro (I.-C.B.); ushefs_cojocaru.marilena@spiruharet.ro (M.C.); ushefs_cojocaru.adin@spiruharet.ro (A.M.C.); 2 Faculty of Psychology and Science Education, University of Bucharest, 050663 Bucharest, Romania; alina.chiracu@fpse.unibuc.ro; 3 Department of Theory and Methodology of Motor Activities, Faculty of Physical Education and Sport, University of Craiova, 200585 Craiova, Romania; marian.cosma@edu.ucv.ro

**Keywords:** mobile health (mHealth), perceived health competence, digital health behavior, depression, anxiety, stress, self-monitoring

## Abstract

**Highlights:**

**What are the main findings?**

- Perceived usefulness of mHealth applications, but not usage frequency, is consistently associated with lower levels of depression, anxiety, and stress through increased perceived health competence.
- Perceived health competence functions as a key psychological mediator linking mHealth engagement to mental health outcomes in a non-clinical adult sample.

**What are the implications of the main findings?**

- mHealth interventions should prioritize features that enhance users’ sense of health competence and perceived usefulness rather than focusing solely on increasing usage frequency.
- Evaluation frameworks for digital health interventions should incorporate psychological process indicators (perceived competence) alongside traditional engagement metrics.

**Abstract:**

**Background**: Mobile health (mHealth) applications are widely used for lifestyle monitoring and health promotion; however, their psychological impact on mental health outcomes remains insufficiently understood. Beyond usage frequency, the perceived usefulness of mHealth tools and underlying psychological mechanisms play a critical role in explaining their association with psychological distress. The present study examined perceived health competence as a potential mediator linking mHealth use to symptoms of depression, anxiety, and stress. **Methods**: This cross-sectional study included 255 adult participants who completed an online survey assessing frequency of mHealth use, perceived usefulness of mHealth applications, perceived health competence (PHCS), and psychological distress (DASS-21). Separate mediation models were tested for depression, anxiety, and stress, with perceived health competence specified as the mediator. Analyses were conducted using bootstrapped mediation procedures. **Results**: Perceived usefulness of mHealth applications was strongly and positively associated with perceived health competence, which in turn was consistently associated with lower levels of depression, anxiety, and stress. Mediation analyses indicated significant indirect effects of perceived usefulness on all three distress outcomes through perceived health competence. In contrast, frequency of mHealth use showed weaker and less consistent associations, with primarily small direct effects and no significant indirect effects through perceived health competence. **Conclusions**: The findings indicate perceived health competence is a plausible explanatory pathway statistically consistent with the association between perceived usefulness and psychological distress. The psychological benefits of mHealth applications appear to depend less on how frequently they are used and more on whether users perceive them as useful and empowering. These results underscore the importance of incorporating psychological process indicators into the design and evaluation of mHealth interventions.

## 1. Introduction

The rapid proliferation of mobile health (mHealth) applications has transformed the way individuals engage in health-related behaviors, monitor symptoms, and manage chronic or preventive care. Advances in mobile technologies have enabled scalable, low-cost, and highly accessible health interventions, positioning mHealth as a central component of contemporary healthcare systems. International organizations and policymakers increasingly promote digital health solutions as tools for improving population health, enhancing self-management, and supporting preventive care [1,2]. As a result, mHealth applications are now widely used not only for physical health monitoring but also for supporting mental health and psychological well-being.

Despite their widespread adoption, the psychological impact of mHealth applications remains incompletely understood. A growing body of research has examined the effectiveness of digital and mobile interventions for mental health outcomes, including symptoms of depression, anxiety, and stress. While some studies report modest beneficial effects, others indicate small or inconsistent associations, highlighting substantial heterogeneity in outcomes [3,4,5]. These mixed findings suggest that mHealth use alone may be insufficient to produce meaningful psychological change and that additional explanatory mechanisms are likely involved.

One limitation of existing research is the predominant focus on usage metrics, such as frequency of app use or type of application, without sufficient consideration of the psychological processes that may help explain the association between mHealth engagement and mental health indicators. Simply engaging with a health application does not guarantee improved well-being; rather, psychological change is more likely to occur when technology use fosters adaptive cognitive, emotional, or motivational processes. Consequently, there is increasing recognition of the need to move beyond descriptive indicators of mHealth use and to examine the psychological mechanisms that may link technology engagement to mental health outcomes [6,7].

From a psychological perspective, constructs related to perceived competence, self-efficacy, and autonomy have long been identified as central determinants of health behavior and mental well-being. Social cognitive theory emphasizes the role of self-efficacy in shaping individuals’ capacity to initiate and maintain health-related behaviors [8], while self-determination theory highlights the importance of perceived competence and autonomy for psychological functioning and well-being [9,10]. In the context of mHealth, applications that support self-monitoring, goal setting, and feedback may enhance users’ sense of agency and competence in managing their health, which in turn may contribute to better mental health outcomes.

Perceived health competence represents a particularly relevant construct in this regard. Defined as individuals’ beliefs in their ability to effectively manage and influence their health, perceived health competence reflects a domain-specific sense of competence and efficacy [11]. Research has shown that higher perceived health competence is associated with greater engagement in health-promoting behaviors, better treatment adherence, and improved psychological well-being [12,13]. Importantly, perceived health competence is not merely a function of objective health knowledge or behavior, but rather captures a subjective appraisal of one’s capability to manage health-related demands.

Within the mHealth literature, perceived health competence is conceptualized as a key psychological mechanism linking technology use to mental health. By providing tools for tracking symptoms, monitoring progress, and receiving personalized feedback, mHealth applications are associated with increased confidence in their ability to manage their health. This increased sense of competence may, in turn, reduce feelings of helplessness and psychological distress, thereby contributing to lower levels of depression, anxiety, and stress. However, despite its theoretical relevance, perceived health competence has received limited empirical attention as a mediator in the relationship between mHealth engagement and mental health outcomes.

The present study addresses this gap by examining the associations among mHealth engagement, perceived health competence, and psychological distress in a non-clinical adult sample. Specifically, the study investigates whether perceived health competence mediates the relationship between mHealth engagement and symptoms of depression, anxiety, and stress. By focusing on a theoretically grounded psychological mechanism, this research aims to contribute to a more nuanced understanding of how and why mHealth applications may influence mental health. Clarifying these mechanisms may inform the design of future mHealth interventions that not only promote engagement but also actively support users’ sense of competence and psychological well-being.

Based on previous theoretical and empirical evidence, the present study tested the following hypotheses.

**H1.**  *Frequency of mHealth use (FRMH) and perceived usefulness of mHealth applications (UTMH) are positively associated with perceived health competence.*

**H2.**  *Perceived health competence is negatively associated with symptoms of depression, anxiety, and stress.*

**H3.**  *Perceived health competence mediates the relationship between mHealth engagement (FRMH and UTMH) and psychological distress (depression, anxiety, and stress).*

## 2. Materials and Methods

### 2.1. Participants and Procedure

Participants were recruited using a convenience sampling strategy. Eligibility criteria included being at least 18 years old and owning a smartphone. Data were collected via an online survey distributed through social media platforms and mailing lists. Prior to participation, all respondents were informed about the purpose of the study, the voluntary nature of participation, and the confidentiality of their responses. Informed consent was obtained electronically from all participants before survey completion.

The final sample consisted of 255 participants of whom 129 were female (51%) and 126 were male (49%). Participants’ ages ranged from 18 to 58 years (M = 27.28, SD = 10.35). Of the total sample, 178 participants were students (70%), 11 were employed part-time (4%), 55 were employed full-time (22%), and 11 were self-employed or freelancers (4%). Regarding marital status, 126 participants were single (49%), while 129 were in a committed relationship (51%).

With respect to self-rated health, five participants reported poor health (2%), 40 reported fair health (16%), and 210 reported good health (82%).

The study was conducted in accordance with the ethical standards of the institutional research committee and the principles of the Declaration of Helsinki (Accord no. 599/14.10.2025, University of Craiova). Participation was voluntary, and no financial or material incentives were offered. All data were collected anonymously and stored securely in accordance with data protection regulations.

### 2.2. Measures

Demographic variables were collected using a brief set of questions assessing participants’ gender, age, occupational status, marital status, and self-rated health (poor, fair, or good).

mHealth Application Types used was assessed using a list of eight items referring to different health-related domains of interest. Participants were asked to indicate how frequently they used mHealth applications for each domain on a 5-point Likert scale ranging from 1 (never) to 5 (very frequently) including step monitoring and heart rate tracking). Before completing this section, participants were provided with a list of example applications to help them more easily identify the mHealth applications they use (Appendix A).

#### 2.2.1. mHealth Engagement

The engagement of mobile health applications was assessed through a brief eight-item self-report questionnaire specifically designed for this study. It included four items evaluating the frequency and intensity of use, including daily use, duration, number of sessions per day, and active days per month and four items measuring the perceived usefulness of health-related applications used (e.g., I feel that health applications help me gain a better understanding of my body). Two mean scores were computed, one for frequency and one for perceived usefulness. The development of the mHealth engagement items followed established principles of psychological measurement. Initially, the constructs of frequency/intensity of use and perceived usefulness were theoretically defined based on prior literature on mHealth engagement and self-regulation. An AI-based language model (ChatGPT, OpenAI-5.2 version) was used as a supportive tool to assist in the generation and refinement of preliminary item wording, under close supervision of the authors. All items were subsequently reviewed, revised, and selected by the authors to ensure conceptual clarity, theoretical alignment, and content validity. To examine the internal structure of the newly developed mHealth engagement items, an exploratory factor analysis (EFA) was conducted using principal axis factoring with oblique Promax rotation, given the expected correlation between dimensions. Sampling adequacy was good (KMO = 0.84), and Bartlett’s test of sphericity was significant (χ^2^(28) = 1161.58, *p* < 0.001). The analysis supported a two-factor solution corresponding to frequency/intensity of use (FRMH) and perceived usefulness (UTMH). The two-factor solution showed strong and theoretically coherent factor loadings. Items assessing frequency/intensity of use loaded strongly on the first factor (λ = 0.65–0.86), while items assessing perceived usefulness loaded primarily on the second factor (λ = 0.49–0.88), with minimal cross-loadings. One item (UT3) showed moderate cross-loading, suggesting partial conceptual overlap between perceived usefulness and engagement intensity, which is theoretically plausible given the nature of mHealth use. Overall model fit was acceptable (RMSEA = 0.08, TLI = 0.96). Internal consistency was good for both subscales (FRMH α = 0.83; UTMH α = 0.85). Additional support for construct validity is provided by the distinct patterns of association observed for the two mHealth engagement dimensions, with perceived usefulness showing stronger and theoretically consistent relationships with perceived health competence and psychological distress than frequency of use.

#### 2.2.2. Perceived Health Competence

Perceived competence over one’s health was measured using the Perceived Health Competence Scale, PHCS [11], consisting of 8 items such as “I am generally able to accomplish the goals I set for my health”). Responses were rated on a 5-point Likert scale (1 = Strongly disagree, 5 = Strongly agree). Higher scores reflect greater perceived competence in managing one’s health.

#### 2.2.3. Depression, Anxiety, and Stress

Emotional distress was measured with the Depression, Anxiety, and Stress Scales [14], which assesses three subscales of psychological symptoms over the past week. Each item is rated from 0 (Did not apply to me at all) to 3 (Applied to me very much).

### 2.3. Data Analysis

Data analyses were conducted using IBM SPSS Statistics 24 [15] and Jamovi GLM medmod module 2.3 [16]. Preliminary analyses included descriptive statistics and Pearson correlation coefficients to examine associations among mHealth use, perceived health competence, and psychological distress variables. Mediation analyses were conducted to test whether perceived health competence mediated the relationship between mHealth use and psychological distress. Mediation was considered statistically significant when confidence intervals did not include zero. Given the cross-sectional nature of the study, the mediation analyses were conducted to test theoretically informed indirect associations rather than causal pathways. The specified mediation models should therefore be interpreted as statistical representations of hypothesized psychological mechanisms, without implying temporal or causal precedence among variables.

## 3. Results

The Results section is organized as follows. First, we report descriptive statistics for mHealth application domains used in the sample. Next, we present descriptive statistics and bivariate correlations among the main study variables. Finally, we report three mediation models testing perceived health competence as a mediator of associations between mHealth engagement dimensions and depression, anxiety, and stress.

### 3.1. Descriptive Statistics

Regarding mHealth engagement, descriptive analyses were conducted for the eight categories of mHealth applications included in the study. Mean scores and standard deviations were calculated for each domain to reflect the average frequency of use (Table 1).

As shown in Table 1, mHealth use in this sample was predominantly oriented toward lifestyle monitoring applications, with physical activity and step monitoring representing the most frequently used category. Moderate engagement was observed for sleep, cardiovascular, and mental health–related applications, whereas applications related to clinical management (e.g., medication adherence, blood glucose monitoring, and telemedicine) were used relatively infrequently. This pattern is consistent with the predominantly non-clinical profile of the sample.

To further characterize the sample and examine relationships among the main constructs, descriptive statistics and bivariate correlation variables are reported in Table 2.

Descriptive statistics and bivariate correlations among study variables are presented in Table 2. Overall, participants reported moderate levels of mHealth engagement and perceived health competence, alongside low to moderate levels of psychological distress. The two mHealth engagement dimensions were moderately correlated, indicating related but distinct aspects of technology use. Perceived usefulness showed a strong positive association with perceived health competence and consistent negative associations with all three distress indicators. In contrast, frequency of mHealth use displayed weaker and less consistent relationships with both perceived health competence and psychological distress, with no significant associations observed for depression, anxiety, or stress. Together, these patterns suggest that the relationship between mHealth engagement and psychological distress may not be direct, providing a rationale for the mediation analyses examining perceived health competence as an underlying psychological mechanism (Hypothesis 3).

Skewness and kurtosis values for all variables fell within acceptable ranges, indicating no substantial deviations from normality and supporting the use of parametric statistical analyses.

### 3.2. Hypotheses Testing

Given that depression, anxiety, and stress represent related but distinct dimensions of psychological distress, separate mediation models were tested for each outcome. In all models, frequency of mHealth use (FRMH) and perceived usefulness of mHealth applications (UTMH) were specified as independent variables, perceived health competence (HSE) as the mediator, and symptoms of depression, anxiety, or stress as outcome variables. Completely standardized coefficients (β) are reported.

Separate mediation analyses were conducted for each outcome variable. Table 3 presents the mediation results for depression, with frequency and perceived usefulness of mHealth use as independent variables, perceived health competence as a mediator, and depressive symptoms as the outcome.

The mediation model for depression (Table 3) indicated that perceived usefulness of mHealth applications was strongly associated with perceived health competence, which in turn was associated with lower depressive symptoms. The indirect effect of perceived usefulness on depression through perceived health competence was significant, whereas the indirect effect of usage frequency was not. Frequency of mHealth use showed a small direct association with depressive symptoms.

The mediation model for anxiety (Table 4) indicated that perceived usefulness of mHealth applications was positively associated with perceived health competence, which in turn was associated with lower anxiety symptoms. The indirect effect of perceived usefulness on anxiety through perceived health competence was significant, whereas the indirect effect of usage frequency was not. Frequency of mHealth use showed a small direct association with anxiety symptoms.

The mediation model for stress (Table 5) indicated that perceived usefulness of mHealth applications was positively associated with perceived health competence, which in turn was associated with lower stress levels. The indirect effect of perceived usefulness on stress through perceived health competence was significant, whereas the indirect effect of usage frequency was not. The frequency of mHealth use showed a small direct association with stress.

Across all three models, perceived health competence consistently mediated the relationship between perceived usefulness of mHealth applications and psychological distress, including symptoms of depression, anxiety, and stress. In contrast, the frequency of mHealth use showed weaker and less consistent indirect effects. Overall, these findings provide partial support for Hypothesis 3, highlighting perceived health competence as a key psychological mechanism linking mHealth engagement—particularly perceived usefulness—to mental health outcomes.

## 4. Discussion

The present study examined the relationships between mHealth engagement, perceived health competence, and psychological distress, with the aim of clarifying the psychological mechanisms through which mHealth engagement may influence mental health. By testing perceived health competence as a mediator between mHealth engagement and symptoms of depression, anxiety, and stress, this study contributes to a more nuanced understanding of how mHealth applications may affect users’ psychological well-being.

Because the data are cross-sectional, the mediation models reflect indirect associations consistent with the proposed mechanism rather than evidence of causality. In addition to examining overall mHealth engagement and its psychological mechanisms, the present study provides a detailed descriptive overview of the types of mHealth applications most frequently used by participants. The results indicate a clear differentiation in usage patterns across application domains. Applications focused on physical activity and step monitoring emerged as the most frequently used, followed by those related to nutrition, weight, and metabolism. These findings suggest that mHealth engagement in this sample is primarily oriented toward lifestyle monitoring and self-management rather than toward clinical or disease-specific purposes.

Moderate levels of use were observed for applications addressing sleep and recovery, cardiovascular monitoring, and mental health and emotional regulation. This pattern indicates that while participants occasionally engage with applications targeting physiological and psychological regulation, these domains are not yet central to their routine mHealth use. In contrast, applications related to medication management, blood glucose monitoring, and telemedicine were used relatively infrequently, which is consistent with the predominantly non-clinical profile of the sample. Together, these findings highlight that mHealth use in the general population is largely preventive and wellness-oriented rather than treatment-focused.

Importantly, these descriptive results provide valuable context for interpreting the mediation analyses. The predominance of lifestyle-related mHealth applications suggests that users are more likely to interact with tools that support daily self-regulation and health awareness. Within this context, the perceived usefulness of mHealth applications appears particularly relevant, as users may derive psychological benefits not simply from using such tools, but from perceiving them as meaningful and supportive in managing everyday health behaviors. This observation reinforces the study’s central finding that perceived health competence functions as a key psychological mechanism linking mHealth engagement to lower levels of psychological distress.

Consistent with the study hypotheses, perceived usefulness of mHealth applications (UTMH) was strongly and positively associated with perceived health competence. In turn, higher perceived health competence was consistently associated with lower levels of depression, anxiety, and stress. Mediation analyses further demonstrated that perceived health competence significantly mediated the relationship between perceived usefulness of mHealth applications and all three dimensions of psychological distress.

In contrast, frequency of mHealth use (FRMH) showed weaker and less consistent associations with perceived health competence and psychological distress. While small direct associations between frequency of use and distress outcomes were observed, the indirect effects of frequency through perceived health competence were not statistically significant. This pattern underscores the distinction between behavioral exposure to mHealth technologies and the subjective appraisal of their usefulness, highlighting the latter as a more meaningful determinant of mental health outcomes.

Importantly, the small but negative association observed between frequency of mHealth use and perceived health competence (β = −0.12) should not be interpreted as a contradiction of the theoretical framework. Rather, this finding may reflect reactive or compensatory patterns of mHealth engagement, in which frequent monitoring is driven by uncertainty, symptom vigilance, or reassurance-seeking rather than by internalized competence. Prior research suggests that excessive health monitoring and self-tracking may increase dependence on external feedback and fail to reduce uncertainty, particularly among individuals with elevated health-related anxiety [17,18]. From this perspective, a higher frequency of mHealth use does not uniformly translate into greater perceived control or competence. This interpretation is consistent with prior research on self-tracking and reassurance-seeking behaviors, suggesting that higher frequency of monitoring does not uniformly translate into greater perceived control or competence.

### 4.1. The Role of Perceived Health Competence as a Psychological Mechanism

The findings align well with theoretical frameworks emphasizing the importance of perceived competence and self-efficacy in health behavior and psychological well-being. According to social cognitive theory, individuals’ beliefs about their capacity to manage health-related challenges play a central role in shaping emotional responses and coping behaviors [8]. Similarly, self-determination theory posits that perceived competence is a basic psychological need, and its satisfaction is essential for optimal functioning and mental health [9,10].

In the context of mHealth, applications that provide feedback, goal tracking, and personalized information may strengthen users’ perceived health competence by fostering a sense of control and mastery over health-related behaviors. The present findings suggest that this enhanced sense of competence may, in turn, buffer against psychological distress. Importantly, these results indicate that mHealth applications do not exert their effects directly on mental health outcomes, but rather operate through users’ psychological interpretations and experiences of technology use.

### 4.2. Frequency of Use Versus Perceived Usefulness

An important contribution of this study lies in differentiating between the frequency of mHealth use and perceived usefulness. While frequency reflects behavioral engagement, perceived usefulness captures the subjective value and meaningfulness of mHealth applications to the user. The observation that perceived usefulness—but not frequency—was consistently linked to perceived health competence and indirectly to psychological distress suggests that “more use” is not necessarily better. Instead, the psychological benefits of mHealth may depend on whether users perceive these tools as genuinely helpful and empowering.

Although a higher frequency of mHealth use is often assumed to reflect greater engagement, the present findings suggest that frequency may capture heterogeneous and potentially maladaptive patterns of use. Prior research indicates that frequent self-monitoring and health tracking can, in some individuals, be driven by health-related anxiety, symptom vigilance, or reassurance-seeking behaviors rather than by proactive self-regulation [17,18,19]. From this perspective, high-frequency mHealth use may reflect reactive or compensatory attempts to regain control in contexts of uncertainty or distress. Such patterns of engagement may increase dependence on external feedback, reinforce uncertainty, and ultimately fail to enhance—or even undermine—perceived health competence. From a self-determination theory perspective, behavioral repetition alone does not guarantee satisfaction of the need for competence, particularly when feedback is not meaningfully internalized or integrated into a coherent sense of agency [9,10]. Accordingly, frequency of mHealth use should not be assumed to represent a linear indicator of effective engagement, but rather a heterogeneous behavioral marker encompassing both adaptive and maladaptive forms of technology use. This distinction has important implications for both research and practice. From a research perspective, it highlights the limitations of relying solely on usage metrics when evaluating the effectiveness of mHealth interventions. From a practical standpoint, it suggests that developers and healthcare providers should prioritize user-centered design features that enhance perceived usefulness and support users’ sense of competence, rather than focusing exclusively on increasing engagement or usage frequency.

While the present findings align with theoretical models emphasizing perceived competence as a key psychological mechanism, prior mHealth research has reported mixed associations between mHealth use and mental health outcomes. Meta-analytic and systematic reviews have documented small and heterogeneous beneficial effects of mHealth interventions for depression and anxiety [3,20]. However, other studies have found weak, null, or highly inconsistent associations between app usage and psychological outcomes, particularly when engagement is indexed primarily by usage frequency [21]. Moreover, intensive self-monitoring and high-frequency use have, in some contexts, been associated with increased distress or maladaptive engagement patterns [17].

The current results help clarify these inconsistencies by demonstrating that not all forms of mHealth engagement operate through the same psychological pathways. Specifically, distinguishing between perceived usefulness and frequency of use may account for previously divergent findings in the literature.

### 4.3. Limitations and Future Directions

Several limitations should be acknowledged. An important limitation of the present study concerns its cross-sectional design, which constrains causal interpretation of the observed mediation effects. Although perceived health competence was modeled as a mediator based on strong theoretical foundations, the temporal ordering of mHealth engagement, perceived health competence, and psychological distress cannot be established within the current design. Accordingly, the mediation models should be interpreted as exploratory and theory-driven, reflecting indirect associations rather than causal mechanisms. Longitudinal and experimental designs are necessary to determine whether changes in mHealth engagement precede changes in perceived health competence and subsequent mental health outcomes. An additional limitation concerns the composition of the sample, which consisted predominantly of students and non-clinical participants reporting generally good self-rated health and low to moderate levels of psychological distress. This sample profile may have influenced both patterns of mHealth use and the observed associations among mHealth engagement, perceived health competence, and psychological distress. Specifically, participants in this sample were more likely to engage with mHealth applications for lifestyle monitoring and wellness purposes rather than for clinical management. As a result, perceived usefulness may play a particularly salient role in shaping perceived health competence in non-clinical contexts, where users rely on mHealth tools primarily for self-regulation and preventive health behaviors. These findings may not generalize to clinical populations or individuals with chronic health conditions, for whom mHealth use may be more closely tied to symptom monitoring, treatment adherence, or medical supervision. In such contexts, frequency of use may reflect necessity rather than voluntary engagement, and the psychological mechanisms linking mHealth use to mental health outcomes may differ. Future research should examine these relationships in more diverse and clinically relevant samples, including individuals with diagnosed mental or physical health conditions, to determine the extent to which perceived health competence operates as a similar or distinct mechanism across populations. Given that 70% of participants were students and the mean age was 27 years, the results primarily reflect mHealth use in young, education-linked populations.

Third, all variables were assessed using self-report measures, which may be subject to recall bias and social desirability effects. Although validated instruments were used for perceived health competence and psychological distress, future research could benefit from incorporating objective indicators of mHealth use, such as log data or usage analytics, to complement subjective assessments. Finally, while the present study focused on perceived health competence as a mediator, other psychological mechanisms—such as autonomy, perceived control, or health-related motivation—may also play important roles and warrant further investigation.

Despite these limitations, the study also has several important strengths. A key strength is the focus on a theoretically meaningful psychological mechanism—perceived health competence—rather than relying solely on descriptive indicators of mHealth use. By distinguishing between frequency of use and perceived usefulness, the study provides a more nuanced understanding of how mHealth engagement relates to mental health outcomes. Additionally, the use of separate mediation models for depression, anxiety, and stress aligns with the multidimensional structure of psychological distress and allows for more precise interpretation of the findings. Together, these strengths enhance the theoretical and practical relevance of the study.

### 4.4. Implications for Practice and Policy

The present findings have important implications for the design, implementation, and evaluation of mHealth interventions. From a practical perspective, the results suggest that mHealth applications are most likely to support mental health when they actively enhance users’ perceived health competence. Accordingly, developers should prioritize features that foster a sense of mastery and control, such as clear and personalized feedback, achievable goal setting, and progress tracking that emphasizes users’ capabilities rather than deficits.

Healthcare professionals and organizations implementing mHealth solutions should be cautious in equating high usage frequency with effectiveness. The findings indicate that encouraging frequent use alone may be insufficient to produce psychological benefits. Instead, interventions should focus on supporting users in perceiving mHealth tools as genuinely useful and empowering. Incorporating brief psychoeducational components or guided onboarding processes that clarify how applications can support health self-management may enhance perceived usefulness and engagement quality.

From a policy perspective, these results highlight the importance of moving beyond engagement metrics when evaluating digital health interventions. Policymakers and funding bodies should consider supporting mHealth programs that demonstrate evidence of positive psychological mechanisms, such as increased perceived competence or self-efficacy, alongside traditional usage indicators. Integrating such criteria into digital health guidelines and quality frameworks may contribute to the development of more effective and user-centered mHealth solutions.

## 5. Conclusions

In conclusion, the present study provides evidence that perceived health competence is a key psychological mechanism linking mHealth engagement to mental health outcomes. The findings emphasize that the psychological benefits of mHealth applications depend less on how frequently they are used and more on whether they enhance users’ confidence in managing their health. By highlighting the mediating role of perceived health competence, this study contributes to a more theoretically grounded understanding of mHealth and offers practical insights for the development of more effective and psychologically meaningful digital health interventions.

## Figures and Tables

**Table 1:** Mean scores and standard deviations for mHealth applications use.

mHealth Application	M	SD
1. Physical activity and step monitoring	3.39	1.55
2. Sleep and recovery	2.23	1.49
3. Heart rate, blood pressure, and oxygen saturation	2.26	1.43
4. Nutrition, weight, and metabolism	2.93	1.55
5. Blood glucose monitoring	1.81	1.16
6. Mental health and emotional regulation	2.27	1.48
7. Medication management and treatment adherence	1.88	1.27
8. Telemedicine and online medical consultations	1.92	1.21

**Table 2:** Descriptive statistics and intercorrelations among study variables.

	M	SD	α	FRMH	UTMH	HSE	DEP	ANX	STR
FRMH	7.17	3.65	0.83	1					
UTMH	11.96	4.34	0.85	0.47 **	1				
HSE	24.40	5.12	0.67	0.15 *	0.52 **	1			
DEP	4.81	4.93	0.87	−0.11	0.06	0.23 **	1		
ANX	5.48	5.22	0.89	−0.07	0.10	0.29 **	0.80 **	1	
STR	6.33	5.31	0.89	−0.11	0.04	0.21 **	0.74 **	0.79 **	1

Note: ** *p* < 0.01, * *p* < 0.05.

**Table 3:** Mediation model for depression.

				95% C.I.				
Type	Effect	Estimate	SE	Lower	Upper	β	z	*p*
Indirect	FRMH ⇒ HSE ⇒ DEP	−0.04	0.02	−0.09	0.01	−0.03	−1.74	0.082
	UTMH ⇒ HSE ⇒ DEP	0.16	0.05	0.07	0.26	0.14	3.29	<0.001
Component	FRMH ⇒ HSE	−0.17	0.08	−0.33	−0.00	−0.12	−2.00	0.045
	HSE ⇒ DEP	0.24	0.07	0.11	0.37	0.25	3.51	<0.001
	UTMH ⇒ HSE	0.68	0.07	0.54	0.82	0.58	9.59	<0.001
Direct	FRMH ⇒ DEP	−0.20	0.09	−0.38	−0.02	−0.15	−2.17	0.030
	UTMH ⇒ DEP	−0.00	0.09	−0.18	0.17	−0.00	−0.04	0.968
Total	FRMH ⇒ DEP	−0.24	0.09	−0.43	−0.06	−0.18	−2.56	0.010
	UTMH ⇒ DEP	0.16	0.08	0.00	0.32	0.14	2.01	0.045

**Table 4:** Mediation model for anxiety.

				95% C.I.				
Type	Effect	Estimate	SE	Lower	Upper	β	z	*p*
Indirect	FRMH ⇒ HSE ⇒ ANX	−0.05	0.03	−0.11	0.00	−0.04	−1.83	0.067
	UTMH ⇒ HSE ⇒ ANX	0.22	0.05	0.11	0.32	0.18	4.07	<0.001
Component	FRMH ⇒ HSE	−0.17	0.08	−0.33	−0.00	−0.12	−2.00	0.045
	HSE ⇒ ANX	0.32	0.07	0.18	0.46	0.32	4.50	<0.001
	UTMH ⇒ HSE	0.68	0.07	0.54	0.82	0.58	9.59	<0.001
Direct	FRMH ⇒ ANX	−0.16	0.10	−0.35	0.03	−0.11	−1.62	0.106
	UTMH ⇒ ANX	−0.01	0.09	−0.20	0.17	−0.01	−0.13	0.894
Total	FRMH ⇒ ANX	−0.21	0.10	−0.41	−0.01	−0.15	−2.11	0.035
	UTMH ⇒ ANX	0.21	0.08	0.04	0.37	0.17	2.45	0.014

**Table 5:** Mediation model for stress.

				95% C.I.				
Type	Effect	Estimate	SE	Lower	Upper	β	z	*p*
Indirect	FRMH ⇒ HSE ⇒ STR	−0.04	0.02	−0.09	0.01	−0.03	−1.72	0.085
	UTMH ⇒ HSE ⇒ STR	0.17	0.05	0.07	0.28	0.14	3.18	0.001
Component	FRMH ⇒ HSE	−0.17	0.08	−0.33	−0.00	−0.12	−2.00	0.045
	HSE ⇒ STR	0.25	0.07	0.10	0.40	0.24	3.38	<0.001
	UTMH ⇒ HSE	0.68	0.07	0.54	0.82	0.58	9.59	<0.001
Direct	FRMH ⇒ STR	−0.20	0.10	−0.40	−0.01	−0.14	−2.02	0.044
	UTMH ⇒ STR	−0.03	0.10	−0.22	0.16	−0.02	−.29	0.775
Total	FRMH ⇒ STR	−0.25	0.10	−0.45	−0.04	−0.17	−2.40	0.016
	UTMH ⇒ STR	0.14	0.09	−0.03	0.31	0.12	1.66	0.098

## Data Availability

The original contributions presented in this study are included in the article. Further inquiries can be directed to the corresponding author. Because this data is considered personal and potentially identifiable, it cannot be made public in order to ensure participant confidentiality and compliance with ethical standards and data protection regulations.

## Abbreviations

The following abbreviations are used in this manuscript:

PHCS	Perceived Health Competence
DASS-21	Psychological distress
FRMH	Frequency of mHealth use
UTMH	Perceived usefulness of mHealth applications

## Appendix A. Examples of mHealth Applications and Devices by Health Domain

1. Physical activity and step monitoring
  (monitors steps, distance, calories burned, heart rate, and daily activity levels)
  Applications: Google Fit, Samsung Health, Apple Health, Fitbit App, Garmin Connect, Huawei Health, Strava, Nike Run Club
  Devices: Fitbit Charge/Inspire/Sense, Garmin Vivosmart/Forerunner, Apple Watch, Xiaomi Mi Band, Huawei Watch Fit
2. Sleep and recovery
  (monitors sleep duration, efficiency, sleep stages—REM, deep, and light sleep—and overall sleep quality)
  Applications: Sleep Cycle, Pillow, Calm, Fitbit Sleep, Oura App, SleepScore, Samsung Health (Sleep section)
  Devices: Oura Ring, Fitbit Sense, Withings Sleep Analyzer, Garmin Venu, Apple Watch
3. Heart rate, blood pressure, and oxygen saturation
  (tracks cardiovascular parameters such as heart rate, blood pressure, heart rate variability, and blood oxygen levels)
  Applications: Qardio, Withings Health Mate, Apple Health, Samsung Health, Cardiio, Omron Connect
  Devices: Apple Watch (ECG/SpO_2_), Samsung Galaxy Watch, Withings BPM Connect, QardioArm, Omron Evolv
4. Nutrition, weight, and metabolism
  (allows logging of food intake, calorie consumption, and monitoring of body weight and BMI)
  Applications: MyFitnessPal, Yazio, Lifesum, Lose It!, Noom, Fitbit Food Log
5. Blood glucose monitoring
  (allows measurement and tracking of blood glucose levels and their association with diet and physical activity)
  Applications: mySugr, GlucoLog, FreeStyle LibreLink, OneTouch Reveal, Contour Diabetes App
  Devices: FreeStyle Libre Sensor, Dexcom G7, OneTouch Verio Flex, Accu-Chek Guide
6. Mental health and emotional regulation
  (provides mindfulness programs, breathing exercises, stress management tools, guided sleep, and CBT-based exercises)
  Applications: Headspace, Calm, Wysa, Insight Timer, MindDoc, BetterSleep, Stoic
  Devices: Muse Headband (EEG-based meditation), Apollo Neuro (calming vibrations)
7. Medication management and treatment adherence
  (supports treatment tracking, medication reminders, and dose logging)
  Applications: Medisafe, Pill Reminder, MyTherapy, CareClinic
  Devices: Smart pillboxes (e.g., AdhereTech, PillDrill)
8. Telemedicine and online medical consultations
  (enables video consultations, automated symptom assessment, and transmission of medical documents)
  Applications: Ada Health, Babylon Health, Doctor Online (Romania), Telios Care, Medicover Virtual Clinic
  Platforms: Doctolib, AlTibbi, Maple, Teladoc

Note: The listed applications and devices were provided as examples to facilitate participants’ recognition of the mHealth tools they use; the list was not intended to be exhaustive.
